# Global Potential Distribution of *Bactrocera carambolae* and the Risks for Fruit Production in Brazil

**DOI:** 10.1371/journal.pone.0166142

**Published:** 2016-11-10

**Authors:** Cesar A. Marchioro

**Affiliations:** Department of Agriculture, Biodiversity and Forest, Campus of Curitibanos, Centre of Rural Science, Universidade Federal de Santa Catarina, Curitibanos, Santa Catarina, Brazil; Louisiana State University, UNITED STATES

## Abstract

The carambola fruit fly, *Bactrocera carambolae*, is a tephritid native to Asia that has invaded South America through small-scale trade of fruits from Indonesia. The economic losses associated with biological invasions of other fruit flies around the world and the polyphagous behaviour of *B*. *carambolae* have prompted much concern among government agencies and farmers with the potential spread of this pest. Here, ecological niche models were employed to identify suitable environments available to *B*. *carambolae* in a global scale and assess the extent of the fruit acreage that may be at risk of attack in Brazil. Overall, 30 MaxEnt models built with different combinations of environmental predictors and settings were evaluated for predicting the potential distribution of the carambola fruit fly. The best model was selected based on threshold-independent and threshold-dependent metrics. Climatically suitable areas were identified in tropical and subtropical regions of Central and South America, Sub-Saharan Africa, west and east coast of India and northern Australia. The suitability map of *B*. *carambola* was intersected against maps of fruit acreage in Brazil. The acreage under potential risk of attack varied widely among fruit species, which is expected because the production areas are concentrated in different regions of the country. The production of cashew is the one that is at higher risk, with almost 90% of its acreage within the suitable range of *B*. *carambolae*, followed by papaya (78%), tangerine (51%), guava (38%), lemon (30%), orange (29%), mango (24%) and avocado (20%). This study provides an important contribution to the knowledge of the ecology of *B*. *carambolae*, and the information generated here can be used by government agencies as a decision-making tool to prevent the carambola fruit fly spread across the world.

## Introduction

In recent years, there has been a growing concern about the threats to biodiversity, food security and human health associated with biological invasions [1]. This is particularly problematic in the case of agricultural and forest pests because their dispersal is facilitated through trade of products [2]. Biological invasions of agricultural and forest pests result in economic losses of billions of dollars worldwide [3]. Dipterans of the family Tephritidae, commonly known as fruit flies, are a representative example of distribution expansion in recent years and how biological invasions may have negative consequences for agriculture and natural environments [4]. The family Tephritidae includes approximately 4000 species from 500 genera [5], of which 1500 fruit fly species feed on fruits and more than 250 species cause significant losses in economic important crops [6]. Fruit flies are found in practically all areas of the world where fruits are cultivated, and the abundance and intensity of attacks in some regions have led to nearly total crop failure [6]. In addition to the direct losses associated with damage to fruits and the costs of pest control or eradication, fruit flies also cause indirect losses resulting from quarantine restrictions imposed by importing countries to prevent the entry and establishment of unwanted species in their territory [4].

The economic losses associated with biological invasions of fruit flies around the world have prompted a major concern among government agencies and farmers with the introduction of the carambola fruit fly, *Bactrocera carambolae* Drew and Hancock, in South America [7]. This species was identified in the continent for the first time in the county of Paramaribo, Suriname, probably introduced through small-scale trade of fruits from Indonesia [8]. In the subsequent years, the pest spread to French Guyana (1989) and more recently to Brazil (1996), where it is considered A2 quarantine pest [7].This indicates that *B*. *carambolae* has potential to spread to other regions of the world where habitat is favourable.

The carambola fruit fly is a polyphagous species that feeds on more than 100 host plants, including several species of economic importance, such as avocado, guava, lemon, mango, orange and papaya among others [7]. Given the availability of host plants, the risks of *B*. *carambolae* spreading throughout the world should be seriously considered, particularly in areas where fruit crops are cultivated in large scale. The carambola fruit fly invasion in these regions may result in serious economic losses. For instance, estimates indicate that the spread of *B*. *carambolae* throughout Brazil may result in an economic loss of US$ 30.7 million in the first year, and approximately US$ 92.4 million after the third year of infestation [7]. In addition to economic losses, there is a growing concern over the negative impacts that the increasing use of chemicals for pest control may cause on the environment [7]. In this context, mapping the areas at risk of invasion by *B*. *carambolae* is an important tool for decision-making aimed at preventing the spread of this pest.

Ecological niche models (ENMs) have been widely used as a decision-making tool in pest risk analysis based on their ability to forecast suitable areas for pest occurrence, allowing the adoption of preventive control measures [9]. Correlative models are the most commonly used approach for this purpose. This method associates species occurrence data with environmental geographic data to generate a suitability gradient that is projected onto a geographic space [10]. In this study, ENM approaches integrated with spatial analysis were used to answer a series of questions involving the potential risks of *B*. *carambolae* spreading across the world. First, environmental geographic data of each known occurrence for the carambola fruit fly was used to compare the climate space occupied by native and invasive populations. Then, correlative ENMs were used to predict the suitable environments available to *B*. *carambolae*. Finally, by integrating ENM and spatial analysis, the extent of the fruit acreage in Brazil that may be at risk of attack by *B*. *carambolae* was assessed. Together, these information may be used by policy makers as a decision tool to prevent the spreading of the carambola fruit fly to the suitable areas, including the major fruit producing areas of Brazil.

## Material and Methods

### Species occurrence data

Records of confirmed presences of *B*. *carambolae* were obtained from the literature, as well as from online databases such as the Global Biodiversity Information Facility (GBIF, http://www.gbif.org/) and SpeciesLink (http://splink.cria.org.br/). When geo-referenced localities were not available (only locality names), geographic coordinates were obtained with the software Google Earth. Overall, 51 unique occurrence points were assembled, of which 36 points were from the native range and 15 points were from invaded areas in South America (Suriname, French Guyana and Brazil–S1 Table). The precise location of all surveyed occurrence were checked using the software Google Earth and only localities within the known distribution range of the species were used for analysis [11]. In order to reduce spatial autocorrelation, these records were submitted to a spatial filtering, delimiting a minimum distance of 10km between each locality data [12,13]. This is greater than the maximum dispersal distance of ≅ 5km reported for *Bactrocera* species, with the majority of individuals recaptured within 1 km from the release point [14, 15]. This procedure was performed using SDMtoolbox [16], resulting in a total of 44 unique occurrence records.

### Environmental data

Current climate data were obtained from the Worldclim database at the resolution of 2.5 arc-min (available at http://www.worldclim.org) [17]. The Worldclim dataset is derived from measurements of monthly temperature and precipitation values collected from weather stations across the world between 1950 and 2000 [17]. The predictor variables employed to assess current climate conditions were selected among nineteen bioclimatic variables that are widely used in studies of ecological niche modelling because they capture annual climatic ranges and limiting factors that are known to influence species geographic distribution [18].

### Climate space occupied by native and introduced populations

Projection of models onto another region relies on the assumption that invasive species conserve their climatic niche in the invaded regions [19]. However, recent studies have demonstrated that species can change their realized climatic niche during invasion [19–21]. A principal component analysis (PCA) was run using all environmental variables to compare the climate space occupied by native and invasive populations of *B*. *carambolae*. A thousand random points from the native and invasive background were added to the PCA analysis (see background selection below), and the first two components were plotted as a biplot, clustering the native and invasive populations with convex hulls to investigate niche overlap within the environmental space [22].

### Ecological niche modelling procedures

ENMs were developed using a maximum entropy algorithm implemented in the software MaxEnt version 3.3.3k [23]. MaxEnt is a general-purpose machine learning software that uses presence-only data [23]. It has been widely used to predict species distribution, including tephritid species [4,22] and in addition to its robust statistical properties, MaxEnt shows a high performance across several niche modelling methods for presence-only data [24].

Building models with an appropriate amount of complexity is critical to avoid over- and under-fitting [25], and produce robust inference [26], particularly when they are transferred to other geographic regions. Model complexity was addressed with the following steps: (i) spatial filtering of occurrence data (as previously described), (ii) using the geographically structured modeling approach [27], (iii) reducing the number of environmental predictors through an *a priori* selection of uncorrelated variables, (iv) delimiting the study area, and (v) tuning experiments through different combinations of feature classes and regulation multiplier values.

A modified *k*-fold cross-validation (commonly called masked geographically structured cross-validation) was employed in the modelling process [27]. Following this approach, occurrence data was partitioned in four groups based on spatial clustering of occurrence points [16], rather than split the data randomly in groups of equal sample size, as the *k-*fold cross-validation implemented in MaxEnt. Models were built using *k–* 1 groups for calibration, and then evaluated with the withheld group. This method provides spatially independent evaluation data, and has been suggested for studies involving the transference of models across space [27,28]. This procedure was performed using SDMtoolbox [16].

Several studies have demonstrated that less complex and robust models can be produced by excluding highly correlated variables, because they do not add new information to the model [29,30], and/or through *a priori* selection of variables based on their biological significance [29]. Here, these two procedures were adopted for variable selection. First, two sets of variables were selected and then the Pearson’s correlation test performed with the software ENMtools v 1.3 [31] was used to ensure the lack of multicolinearity among the selected predictors [32]. The first set of predictors was selected based on previous studies that successfully modeled the distribution of other *Bactrocera* species [4,22] and included the following climatic variables: annual mean temperature (Bio1), mean diurnal range (Bio2), maximum temperature of warmest month (Bio5), minimum temperature of coldest month (Bio6), annual precipitation (Bio12), precipitation of wettest (Bio13) and driest month (Bio14). Additionally, a second set of variables was employed by removing the variables Bio5 and Bio6.

**Fig 1:**
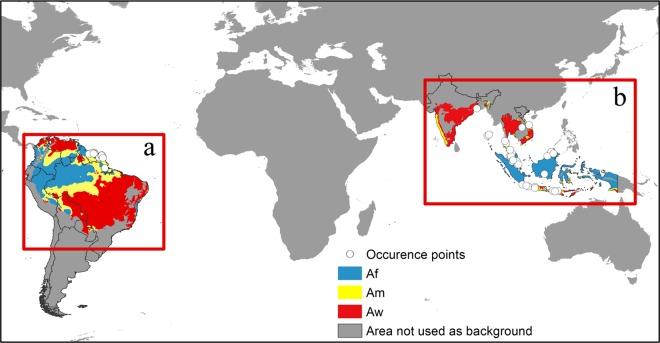
Background and occurrence points of native (b) and invasive (a) populations of *Bactrocera carambolae* used in the modeling process. Colours refer to Köppen-Geiger classifications in the native and invaded range, while the grey area represents areas not used as background. *Af =* extremely hot and moist; *Am* = extremely hot and xeric; *Aw* = extremely hot and arid.

MaxEnt and most correlative ENMs generate pseudo-absence sample points randomly selected from the background study area [33]. While some studies used a minimum convex polygon around the occurrence points as background, others adopted a less arbitrary selection based on biophysical classifications such as biomes [34] or climatic zones [22,35]. Here, bioclimatic methods of background selection were adopted given their simplicity and practicality and because they have proved to be effective for other fruit fly species [22]. The distribution records were intersected with Köppen-Geiger climate zones obtained from CliMond (http://www.climond.org) at the spatial resolution of 2.5 arc-minutes. The climate zones containing one or more distribution records were used to restrict background during model training (Fig 1).

MaxEnt allows users to select a variety of “feature classes” that can be used to build very complex and highly nonlinear response curves [26]. A feature is a function of an environmental variable, and in MaxEnt it can be a combination of six classes: linear (L), quadratic (Q), product (P), hinge (H) and threshold (T). Because parsimonious models can be generated using different combinations of feature classes [36–38], five of these combinations were tested in this study: L; H; LQ; LQH and LQHPT.

While users can specify the feature to be used, MaxEnt automatically selects individual features for each predictor that contribute most to model fit using regularization coefficient β [23,26]. The regularization coefficient can be tuned by multiplying it by a user-specified constant (Regularization multiplier), altering the overall level of regularization rather than changing the β parameter individually [26,27]. Studies have demonstrated that less complex and transferable models can be built by tuning the regularization multiplier to values higher than the default of MaxEnt [26, 27,38]. Therefore, in addition to MaxEnt default, regularization multiplier values of 3 and 5 were also tested in the development of the models.

### Model evaluation

The performance of the models was assessed using threshold-independent and threshold-dependent metrics. As threshold-independent evaluation, the Area Under the Curve (AUC) and the Bayesian Information Criterion were used (BIC). For presence-background evaluations, AUC assess the discriminatory ability of the model, quantifying the probability that the model correctly ranks a random presence locality higher than a random background pixel [27]. AUC values range from 0 to 1; a value of 0.5 indicates the model did not perform better than random; values between 0.5 and 0.7 indicate poor performance; between 0.7 and 0.9 indicate reasonable or moderate performance; while values higher than 0.9 indicate high performance [10]. Additionally, BIC was calculated with the software ENMtools v1.3 [31] using the full data set. BIC provides information on the relative quality of a model taking into account model complexity (number of parameters) and goodness-of-fit [25].

Two threshold-dependent metrics were used to evaluate model performance: omission rates (OR) at the minimum training threshold (MPT), and OR at 10% training presence threshold (TP10). The expected value of OR at MTP is 0 and 0.10 at TP10. Values higher than the expected indicate over-fitting and poor performance of the models [13,28]. In order to select models with high performance and low complexity, the following criteria was adopted: OR at MTP and TP10 closer to 0 and 0.10, respectively, low BIC values and AUC values higher than 0.8.

### Model transfer across space

Once the best model was selected, it was projected onto other regions of the world to access the global potential distribution of *B*. *carambolae*. Because models are calibrated based on the relationship between occurrence records and climate in the study area, projecting it onto other regions with non-analogous climatic conditions can be problematic, since the models are not informed about how species would respond to climatic novelty [39,40]. To assess climate novelty in the transferred regions, a Multivariate Environmental Similarity Surface test (MESS) implemented in MaxEnt was performed. MESS is an index that expresses the similarity of any given point to a reference set of points, with respect to the chosen predictor variables [32]. Negative values discriminate areas where at least one variable has a value that is outside the calibration range. Additionally, we restricted model projections to climate conditions encountered during training by disabling extrapolate options in MaxEnt. The final model was run with the logistic output and then binary maps displaying unsuitable and suitable environments were built using the thresholds MTP and TP10. Areas above the MTP were referred as suitable, whereas areas above TP10 were considered optimal for *B*. *carambolae* [41].

### Spatial analyses

A survey in the CABI database (www.cabi.org/isc/datasheet/8700) was conducted to assess the plants used as host by *B*. *carambolae*. Based on this survey, the acreages of the following economically important fruit species were obtained for each Brazilian municipality in 2014 from the database of the Brazilian Institute of Geography and Statistics (IBGE, available at www.ibge.gov.br): avocado, cashew, guava, orange, lemon, papaya, mango and tangerine (S1 Fig). In order to quantify the production areas at risk of attack, the suitability map of *B*. *carambola* were intersected against maps of fruit acreage in Brazil. Municipalities that partially or totally overlapped with the predicted distributions of the carambola fruit fly were accounted as at risk of attack. Both the acreage and the number of municipalities at risk of attack by *B*. *carambolae* were assessed.

## Results

### Climate space occupied by native and introduced populations

**Fig 2:**
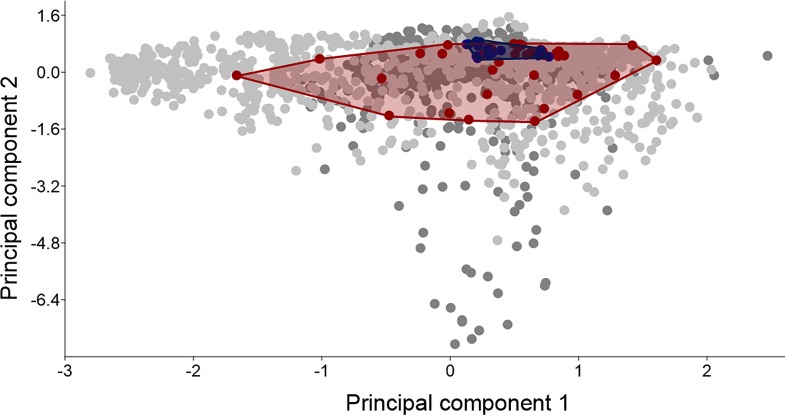
Principal component analysis (PCA) performed with 19 bioclimatic variables. Red symbols represent native populations; blue symbols are invasive populations, light and dark grey points represent 1000 random points extracted from the native and invasive backgrounds, respectively.

The carambola fruit fly is found across three and two Köppen-Geiger climatic zones in its native and invaded ranges, respectively. In both native and invaded ranges, *B*. *carambolae* populations predominantly occur in tropical zones with extremely hot and moist regions (S2 Fig). The principal component analysis of the climatic data defined a climate space of reduced dimensionality allowing the investigation of niche conservatism and differentiation. The first two components of the PCA were significant and together explained 70.3% of the overall variation. The principal component analysis showed a high niche overlap between native and introduced populations (Fig 2). The accessible climate space available to *B*. *carambolae* in its native and invaded ranges (light and dark grey points in Fig 2) forms two overlapping clouds, and indicates that the accessible climate space in the invaded range includes only a portion of the climate space occupied in the native region.

### Model assessment

Overall, 30 MaxEnt models built with different combinations of environmental predictors, feature classes and regularization were evaluated for predicting the potential distribution of the carambola fruit fly (Table 1). Although all models performed better than random, AUC values varied widely, ranging from 0.61 to 0.92. This variation in model performance can also be observed by the comparison between observed and expected omission rates at the thresholds MTP and TP10. Some models showed omission rates of up 0.31 above the expected value. In general, higher AUC and lower omission rates and BIC values were obtained as regularization multiplier increased. The models built using the default settings of MaxEnt showed the highest omission rates, indicating model over-fitting. Based on the criteria adopted in the present study, the best model included seven variables (Bio1, Bio2, Bio5, Bio6, Bio12, Bio13, Bio14), linear, quadratic and hinge features, regularization multiplier equals to 5, and showed the lowest BIC value and omission rates, as well as AUC higher than 0.9.

**Table 1:** Summary of performance statistics of models for *Bactrocera carambolae*. Best models are highlighted in bold, and the asterisk indicates the selected one.

MaxEnt settings			BIC	ΔBIC	AUC	Omission rate	
Variables^1^	Features	RM				MPT	10%
Bio1, Bio2, Bio5, Bio6 Bio12, Bio13, Bio14	L	1	982.20	8.95	0.81	0.11	0.28
		3	980.28	7.04	0.89	0.11	0.25
		5	977.38	4.14	0.92	0.11	0.14
	H	1	1017.61	44.36	0.76	0.27	0.33
		3	978.27	5.03	0.91	0.02	0.17
		5	980.89	7.65	0.90	0.11	0.12
	LQ	1	980.51	7.27	0.84	0.11	0.25
		3	976.85	3.61	0.89	0.06	0.16
		**5**	**973.52**	**0.28**	**0.90**	**0.04**	**0.14**
	LQH	1	1005.56	32.32	0.73	0.18	0.36
		3	985.47	12.23	0.89	0.04	0.20
		5*	**973.24**	**0.00**	**0.91**	**0.02**	**0.14**
	LQHPT	1	999.15	25.90	0.76	0.18	0.41
		3	980.92	7.68	0.89	0.04	0.22
		5	979.74	6.50	0.88	0.07	0.18
Bio1, Bio2, Bio12, Bio13, Bio14	L	1	978.63	5.39	0.70	0.11	0.30
		3	980.34	7.09	0.75	0.11	0.25
		5	977.38	4.14	0.84	0.07	0.16
	H	1	1007.18	33.93	0.73	0.09	0.27
		**3**	**975.17**	**1.93**	**0.84**	**0.02**	**0.16**
		5	980.89	7.65	0.86	0.02	0.14
	LQ	1	980.49	7.25	0.73	0.07	0.30
		3	976.85	3.61	0.70	0.07	0.29
		**5**	**973.95**	**0.71**	**0.86**	**0.05**	**0.14**
	LQH	1	998.45	25.20	0.70	0.05	0.21
		3	985.47	0.89	0.80	0.05	0.20
		**5**	**973.83**	**0.59**	**0.86**	**0.03**	**0.14**
	LQHPT	1	1004.47	31.22	0.61	0.16	0.39
		3	974.13	0.89	0.75	0.09	0.25
		5	1	6.50	0.82	0.03	0.18

^1^Bio1 = Annual mean temperature, Bio2 = Mean diurnal range; Bio 5 = Maximum temperature of warmest month; Bio 6 = Minimum temperature of coldest month; Bio12 = Annual precipitation; Bio13 = Precipitation of wettest month and Bio14 = Precipitation of driest month.

Mean diurnal temperature range (Bio2), precipitation of the driest (Bio14) and wettest month (Bio 13) were the most important predictors that contributed to the final model. The probability of presence decreased as mean diurnal temperature range and precipitation in the driest month increased. By contrast, higher precipitation in the wettest months increased the probability of occurrence of *B*. *carambolae* (S3 Fig).

### Potential distribution of *B*. *carambolae*

**Fig 3:**
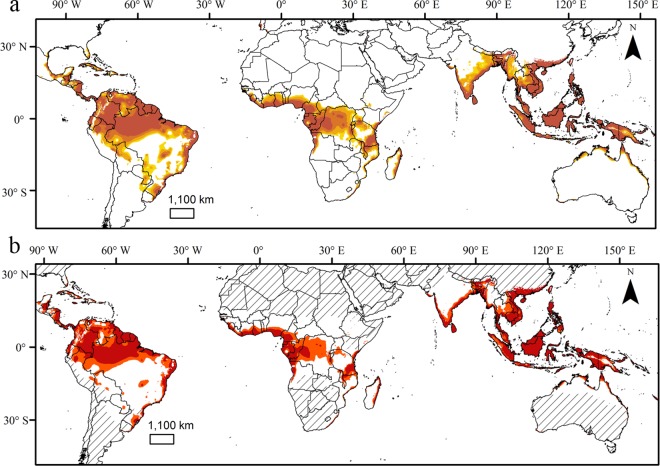
Predicted suitable habitats for *B*. *carambolae* showing logistic (a) and binary outputs (b), as well as MESS analysis (Elith et al. 2010). Warmer colors in the logistic map indicate high suitability. The binary outputs include suitable and optimal conditions for the species, represented by the minimum presence threshold (MTP) and 10% training presence threshold (TP10), respectively.

Potential distribution maps with logistic and binary outputs showing suitable (MTP) and optimal (TP10) conditions are shown in Fig 3. Climatically suitable areas were predicted in Central and South America, Sub-Saharan Africa and Southeast Asia. In Central America, these areas included Costa Rica, Guatemala, Honduras, Panama and eastern coast of Mexico. In South America, suitable areas were identified in Brazil, Colombia, Ecuador and Venezuela within the Amazon region, east coast and isolated areas in Midwest and Southern Brazil, Southern Paraguay and Northern Argentina. Optimal areas for the occurrence of *B*. *carambolae* included Amazon region and east cost of Brazil.

In Africa, suitable habitats were predicted in sub-Saharan countries within monsoon, tropical rainforest and tropical savanna climate conditions, including Democratic Republic of Congo, Congo, Gabon, Equatorial Guinea, west of Angola and Southern Nigeria, Benin, Togo, Ghana, Ivory Coast, Liberia and Sierra Leone, northern and southeastern Tanzania, Southeast Kenya and east coast of Mozambique and Madagascar (Fig 3). Areas predicted as optimal for *B*. *carambolae* included western sub-Saharan Africa and Southeastern Democratic Republic of Congo and east coast of Mozambique and Madagascar. Projection also indicates that areas in east and west coast of India and northern Australia are under risk of invasion by *B*. *carambolae*.

The areas predicted as suitable for *B*. *carambolae* include mostly regions within the climate categories of Köppen-Geiger found in its native range, characterized by hot and humid climates (*Af* and *Am*) or extremely hot and arid areas (*Aw*). This indicates that suitable habitats typically exhibit a high annual precipitation, but not necessarily throughout the year, similarly to that found for *B*. *invadens* [4]. The areas predicted as suitable in Southern Brazil and China, which are classified as humid subtropical climate, reinforce these findings, since these regions are characterized by warm summers with precipitation dispersed throughout the year (*Ca* and *Cb*).

MESS analysis [32] indicating climatic areas outside the model’s calibrated range (non-analogous climates) are shown in Fig 3. These areas included southwestern South America, northern and southern Africa and central and southern Australia. An examination of the most dissimilar variables shows that the differences observed between transferred areas and those used for calibration are associated with maximum and minimum temperatures and annual precipitation.

### Risks to Brazilian fruit production

**Fig 4:**
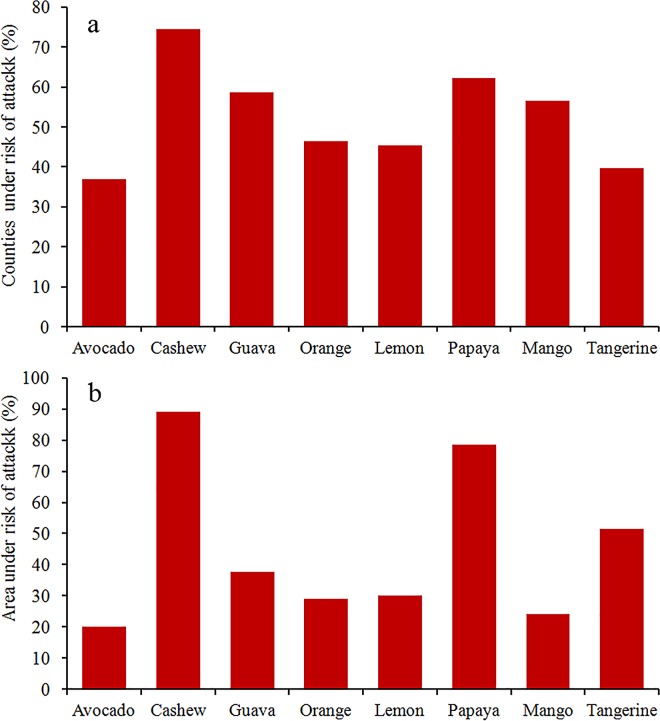
Percentage of counties and acreage of eight economically important fruit species cultivated in Brazil under risk of attack by *Bactrocera carambolae*.

The acreage of eight economically important fruit species under risk of attack varied widely, which is expected because the production of these fruits is concentrated in different regions of the country (Fig 4). The production of cashew is the one that is at higher risk, with almost 90% of its production area within the suitable range of *B*. *carambolae*, followed by papaya (78%), tangerine (51%), guava (38%), lemon (30%), orange (29%), mango (24%) and avocado (20%). In cases such as guava, mango and orange, the percentage of municipality within the potential distribution range of *B*. *carambolae* is higher than the percentage of producing area under risk. This indicates that major producing areas of these fruits are outside the potential distribution range of the carambola fruit fly.

## Discussion

This study integrated ENM methods and spatial analysis to identify climatically suitable areas for the occurrence of *B*. *carambolae* in a global scale, focusing on the risks of invasion in the major fruit production areas in Brazil. Prior to the development of the model, niche conservatism was evaluated based on known native and invaded occurrences and the results indicate that no significant climatic niche shift occurred during the invasion of South America. Also, the comparison between the niche occupied by native and invaded populations indicates that the accessible climate space in the invaded range includes only a portion of the climate space occupied in the native region, suggesting that *B*. *carambolae* may expand its actual range in South America if effective preventive measures are not taken.

The performance of the models varied widely as a result of the changes made in the default settings of MaxEnt. In this study, the factors that exert most influence on model performance were regularization multiplier values, followed by the feature classes included in the model and the number of environmental variables. The models ran with the default settings had comparatively poor performance. This indicates the importance of testing different MaxEnt configurations to obtain high-performance models, corroborating the findings of studies conducted with other species [27,37,38,42,43]. Also, by changing the default settings of MaxEnt one can build models with an appropriate level of complexity, which is a desirable attribute for improving model’s transferability to other regions [43].

Selection of predictor variables has been recognized as an essential step in the modelling process [44–46]. Biological significance of the environmental data, resolution, extent of the study range as well as multicollinearity have been cited as factors influencing model results [29, 45, 47, 48]. Here, the recommendation that priority should be given to predictors with biological significance [29,49] was followed and two sets of variables were used. Nevertheless, only subtle differences in model performance were recorded between the two sets of variables. Although AUC values were consistently higher when models were run using seven variables instead of five, the threshold-dependent metrics do not indicate that these models performed better. This corroborates other studies that question the exclusive use of AUC values for model comparison and selection [25,50–53].

The combination of different feature classes may generate highly nonlinear response curves and very complex and over-fitting models [26]. In this context, one can expect that models built using fewer feature classes result in less complex models with better performance. However, the results obtained with different species are conflicting; while some studies found that the default settings generates high performance models [54], others found that forcing the models to use less features resulted in robust models [27,38]. Former studies have shown that an appropriate level of complexity is necessary to correctly model the species response to environmental factors [26,38]. Here, the appropriate level of complexity was obtained by combining complex features (LQH) with higher regularization. This indicates the importance of testing different regularizations to obtain robust models, particularly when projected to other areas, as previously demonstrated by other studies [27, 28, 37, 38, 54].

Prior to inferring areas of potential invasion, it is worth to emphasize that MaxEnt model was used in this study to identify suitable climate space for *B*. *carambolae*, but without consideration of biological interactions and historical factors related to the species’ geographic distribution [10,35]. Particularly in the case of fruit flies, interspecific competition between exotic and native species seems to play an important role on their abundance and distribution [55]. This was demonstrated for *Bactrocera* and other fruit fly species, and interestingly when different *Bactrocera* species invaded new regions previously occupied by polyphagous fruit flies of another genus, the interspecific competition has generally resulted in a reduction in numbers or niche differentiation of the established species [55]. In addition to climate suitability, it is known that propagule pressure (i.e. the number of individuals introduced to a novel region) influences the likelihood of establishment of an insect species in a new geographic area [3,56]. The propagule pressure depends on the frequency and amount of fruits transported from infested regions and the likelihood of these fruits being infested with *B*. *carambolae*.

Because of the above-mentioned limitations, ENMs should be interpreted as the geographical representation of the environmental conditions that are suitable for a species [57,58]. In this context they are an essential tool to identify suitable areas for invasive species, which ultimately represent regions that are more vulnerable to invasion than one presenting unsuitable conditions [4]. According to model predictions, a significant portion of the Brazilian territory was identified as suitable for *B*. *carambolae*, including east coast, northern region and some areas of Midwest and Southern Brazil. Several cultivated and wild plant species were identified as potential hosts for *B*. *carambolae* in South America [59,60]. Some of these species have wide geographical distribution in the region, and may act as corridor of plants, facilitating the spread of the insect pest through the continent.

The spread of *B*. *carambolae* to the areas identified as suitable for the pest may represent a significant economic loss for producers, since that more than a half of the acreages of crops such as cashew, papaya and tangerine are within these areas. Severe economic losses can also occur when only a portion of the acreage of an economically important crop is attacked. This can be the case of orange, whose Brazilian production comprises almost 35% of the world production [61] and 29% of its acreage are within the potential distribution range of *B*. *carambolae*.

In addition to economic losses, the global spreading of *B*. *carambolae* may have high social and environmental costs. Although the economic losses associated with the *B*. *carambolae* invasion of the major fruit producing areas can be quantified, it is difficult to predict the potential impacts on the environment resulted from an increase in the use of pesticides for pest control. Recent experiences with the introduction of insect pests in Brazil show that the indiscriminate use of highly toxic pesticides is a real threat to environment and human health. The bollworm, *Helicoverpa armigera* (Hübner) (Lepidoptera: Noctuidae), was reported for the first time in the South American continent in Brazil in 2013 [62,63], causing an economic damage of approximately US$ 800 million [64]. This situation has led to excessive use of insecticides even when pest populations were low or non-existent, worsening the situation due to the elimination of natural enemies and increase in environmental contamination [64]. Given the recent history of biological invasions of insect pests and the potential losses associated with outbreaks of *B*. *carambolae*, it would not be surprising if the same problems experienced in the recent past reoccur with the carambola fruit fly.

It is widely accepted that preventing invasions is more cost-effective than eradicating or controlling the invading species once they have established in a region [65,66]. The information generated here can be used to the development of pest risk analysis by policy maker and/or plant protection organizations to determine priority areas for sanitary inspection and installment of detection traps. Using the information on fruit trade across the country and the knowledge on the existence of plant corridors associated with the suitability maps generated here, efforts can be coordinated and concentrated strategically in the areas under risk of invasion in order to prevent the spreading of the pest beyond the currently occupied areas.

In conclusion, the present study used occurrence records associated with climatic data to compare the climatic space occupied by native and invasive populations of *B*. *carambolae* and employed ENMs to forecast the suitable habitats available for this species in a global scale. These data were used to estimate the percentage of the fruit acreage at risk of attack by this pest in Brazil. The area currently occupied by *B*. *carambolae* in its introduced range is climatically similar to the native range. Climatically suitable areas were predicted in Central and South America, in Sub-Saharan Africa and in India and Southern China. Because the production of fruits is concentrated in different regions of Brazil, the acreage under risk of attack by *B*. *carambolae* varied widely according to fruit species. The production of cashew is the one that is at higher risk, with almost 90% of its production area within the suitable range of *B*. *carambolae*, followed by papaya and tangerine. This study provides an important contribution to the knowledge on the ecology of *B*. *carambolae* and the data generated here could be used to help direct further experiments and modeling exercises to develop tools for predicting the potential spread and impact of the pest.

## Supporting Information

S1 Fig Maps of fruit acreage built using data from each Brazilian municipality obtained from the Brazilian Institute of Geography and Statistics (Instituto Brasileiro de Geografia e Estatística, IBGE). a–avocado, b–cashew, c–guava, d–lemon, e–mango, f–orange, g–papaya, h–tangerine.
(TIF)

S2 Fig Proportion of native and invasive occurrences as a function of Köppen-Geiger climatic zones. *Af =* extremely hot and moist; *Am* = extremely hot and xeric; *Aw* = extremely hot and arid.
(TIF)

S3 Fig Response curves showing the relationship between predicted probability of presence and bioclimatic variables. (TIF)

S1 Table Occurrence records of *Bactrocera carambolae* used in the study. (DOCX)
