# Supplementary figures and images for: Global Potential Distribution of Bactrocera carambolae and the Risks for Fruit Production in Brazil
Source: PLoS One. 2016 Nov 10;11(11):e0166142. doi: 10.1371/journal.pone.0166142 (PMC5104352; doi:10.1371/journal.pone.0166142)

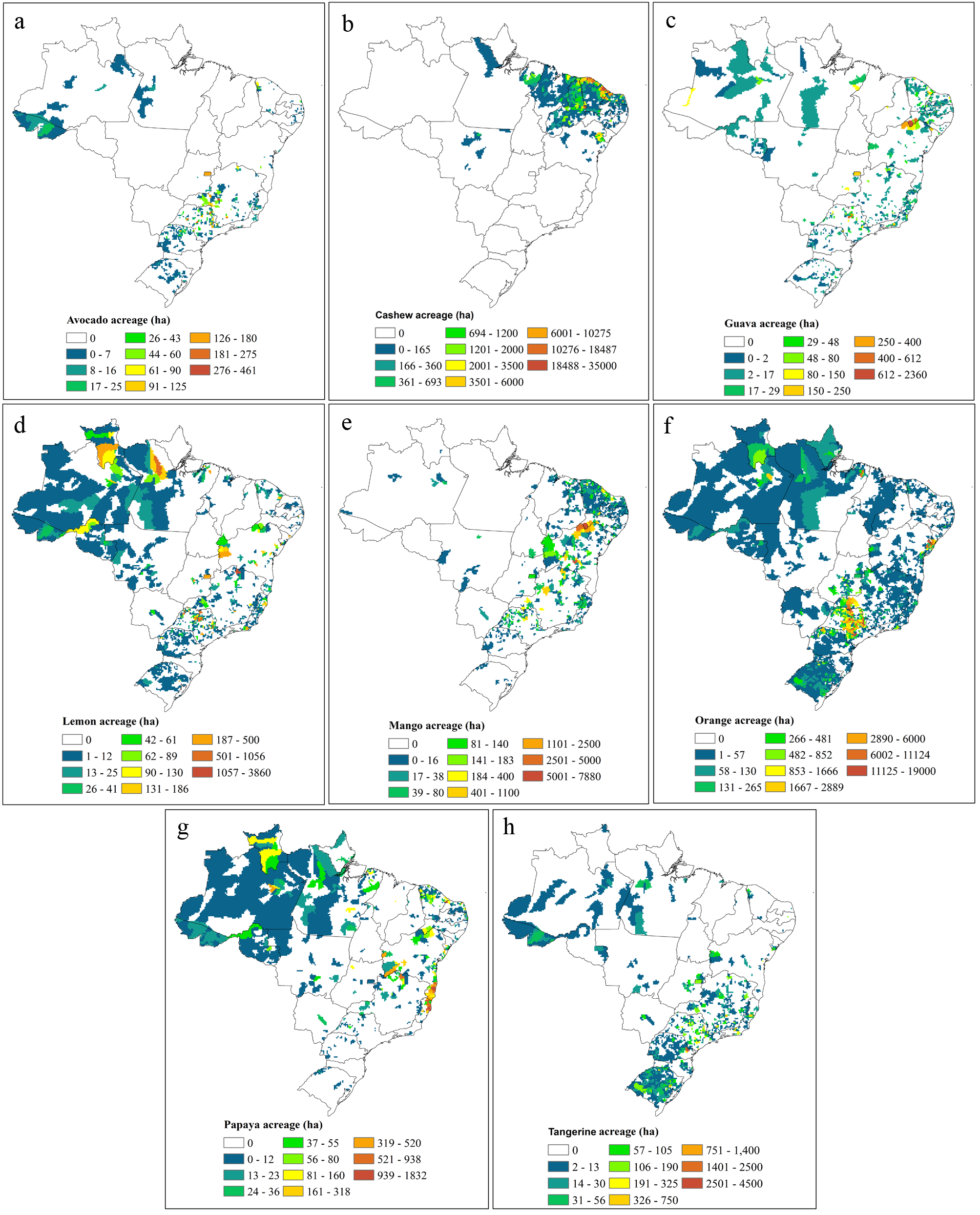

Supplement: S1 Fig — a–avocado, b–cashew, c–guava, d–lemon, e–mango, f–orange, g–papaya, h–tangerine. (TIF) [file pone.0166142.s001.tif]

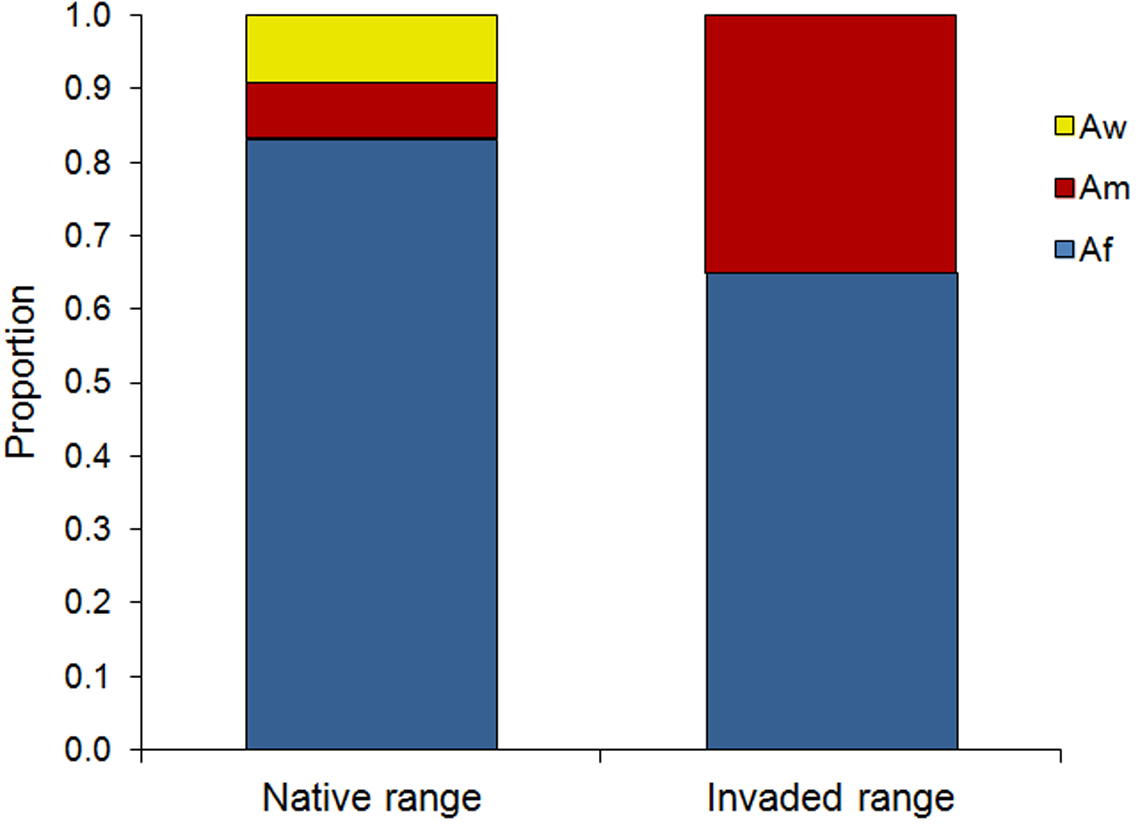

Supplement: S2 Fig — Af = extremely hot and moist; Am = extremely hot and xeric; Aw = extremely hot and arid. (TIF) [file pone.0166142.s002.tif]

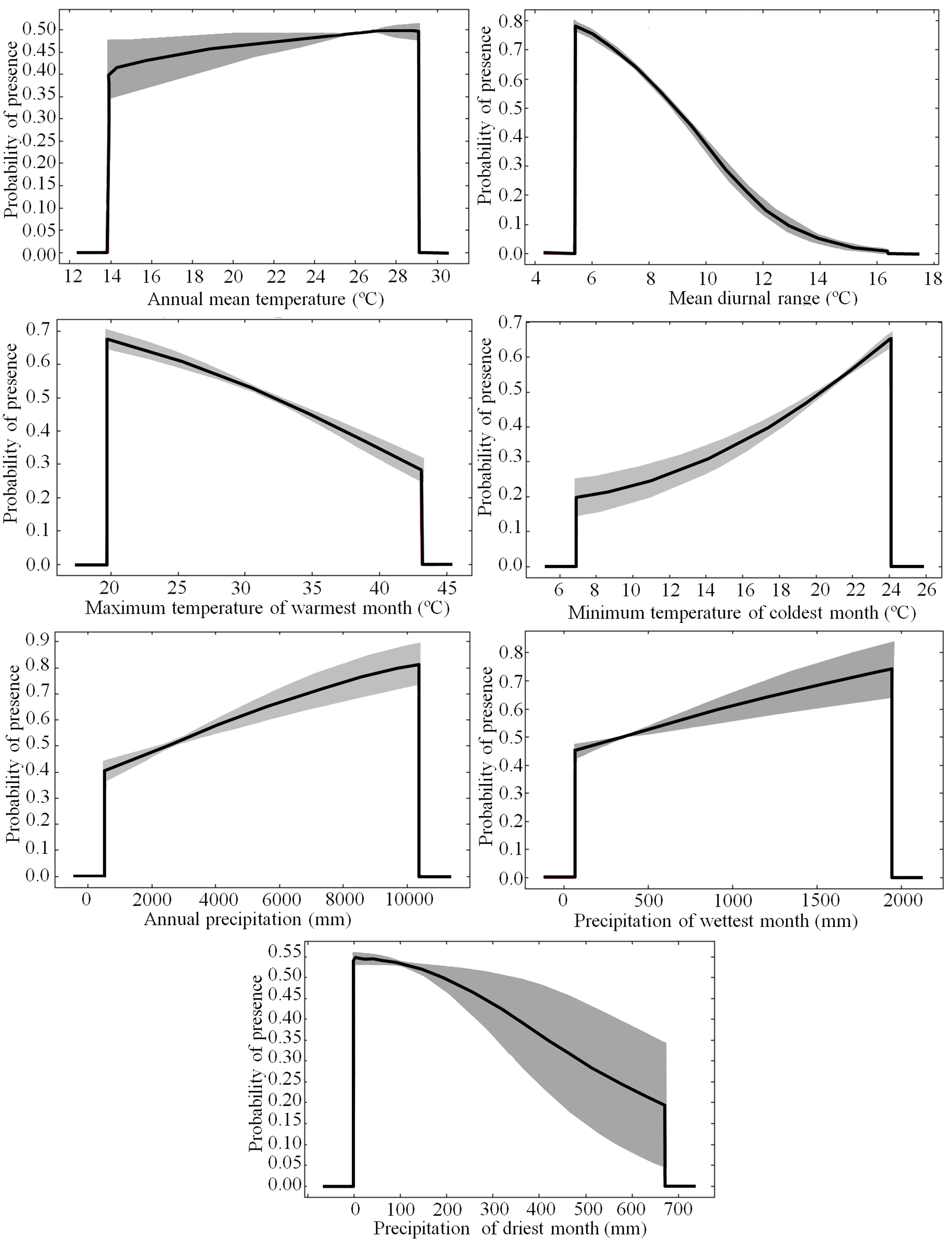

Supplement: S3 Fig — (TIF) [file pone.0166142.s003.tif]
