# Supplementary material for: Global Potential Distribution of Bactrocera carambolae and the Risks for Fruit Production in Brazil
Source: PLoS One. 2016 Nov 10;11(11):e0166142. doi: 10.1371/journal.pone.0166142 (PMC5104352; doi:10.1371/journal.pone.0166142)
Supplement: S1 Table — (DOCX) [file pone.0166142.s004.docx]

Table 1S. Occurrence records of *Bactrocera carambolae* used in the study.

| Latitude | Longitude | Locality | Population |
| --- | --- | --- | --- |
| -8.740 | 117.533 | Sumbawa Regency, Indonesia | Native |
| -8.511 | 118.648 | Bontokape, Indonesia | Native |
| -8.500 | 115.489 | Lombok, Indonesia | Native |
| -7.492 | 110.004 | Wonosobo Regency, Java | Native |
| -7.000 | 110.000 | Gringsing, Batang Regency, Indonesia | Native |
| -6.910 | 107.610 | West Java, Indonesia | Native |
| -6.598 | 106.799 | Bogor, Indonesia | Native |
| -2.190 | 113.540 | Palangkaraya, Indonesia | Native |
| 1.349 | 103.837 | Pierce Lake, Singapore | Native |
| 2.500 | 103.200 | Labis, Malaysia | Native |
| 2.967 | 101.867 | Selangor Malaysia | Native |
| 3.030 | 101.750 | Balakong, Malaysia | Native |
| 3.104 | 101.661 | Kuala Lumpur, Malaysia | Native |
| 3.200 | 101.300 | Kapar, Malaysia | Native |
| 3.900 | 101.410 | Trolak, Malaysia | Native |
| 4.020 | 101.010 | Teluk Intan, Malaysia | Native |
| 4.500 | 114.667 | Belait, Brunei Darussalam | Native |
| 4.520 | 115.000 | Limbang, Malaysia | Native |
| 4.583 | 101.083 | Ipoh, Malaysia | Native |
| 5.450 | 116.000 | Tambunan, Malaysia | Native |
| 5.500 | 117.000 | Kinabatangan, Malaysia | Native |
| 5.543 | 101.342 | Belum Perak, Malaysia | Native |
| 6.080 | 116.160 | Kota Kinabalu, Malaysia | Native |
| 6.833 | 100.667 | Songhkla, Thailand | Native |
| 9.300 | 98.990 | Tha Chang, Thailand | Native |
| 10.100 | 98.750 | Ranong, Thailand | Native |
| 12.150 | 92.750 | Andaman Islands | Native |
| 15.167 | 105.167 | Ubon Ratchathani, Thailand | Native |
| 16.167 | 107.833 | Thua Thien Hue, Vietnam | Native |
| 22.340 | 88.220 | Abhirampur, India | Native |
| 0.000 | -51.283 | Mazagão, Brazil | Invasive |
| 0.000 | -51.167 | Santana, Brazil | Invasive |
| 3.817 | -51.886 | Sant-Georges, French Guiana | Invasive |
| 3.883 | -51.833 | Sant-Georges, French Guiana | Invasive |
| 4.000 | -53.250 | Saül, French Guiana | Invasive |
| 4.317 | -52.133 | Regina, French Guiana | Invasive |
| 4.583 | -52.467 | Roura, French Guiana | Invasive |
| 4.733 | -52.333 | Roura, French Guiana | Invasive |
| 5.200 | -57.167 | Apoera, Suriname | Invasive |
| 5.217 | -57.183 | Apoera, Suriname | Invasive |
| 5.417 | -55.333 | Para, Suriname | Invasive |
| 5.583 | -56.250 | Coronie, Suriname | Invasive |
| 5.667 | -55.583 | Saramacca, Suriname | Invasive |
| 5.867 | -55.167 | Paramaribo, Suriname | Invasive |
